# Metastatic Basal Cell Carcinoma: A Biological Continuum of Basal Cell Carcinoma?

**DOI:** 10.1155/2012/157187

**Published:** 2012-12-06

**Authors:** Karaninder S. Mehta, Vikram K. Mahajan, Pushpinder S. Chauhan, Anju Lath Sharma, Vikas Sharma, C. Abhinav, Gayatri Khatri, Neel Prabha, Saurabh Sharma, Muninder Negi

**Affiliations:** ^1^Department of Dermatology, Venereology and Leprosy, Dr. RP Government Medical College, Kangra, Tanda 176001, Himachal Pradesh, India; ^2^Department of Pathology, Dr. RP Government Medical College, Kangra, Tanda 176001, Himachal Pradesh, India; ^3^Department of Radiotherapy and Oncology, Dr. RP Government Medical College, Kangra, Tanda 176001, Himachal Pradesh, India

## Abstract

Basal cell carcinoma (BCC) accounts for 80% of all nonmelanoma skin cancers. Its metastasis is extremely rare, ranging between 0.0028 and 0.55 of all BCC cases. The usual metastasis to lymph nodes, lungs, bones, or skin is from the primary tumor situated in the head and neck region in nearly 85% cases. A 69-year-old male developed progressively increasing multiple, fleshy, indurated, and at places pigmented noduloulcerative plaques over back, chest, and left axillary area 4 years after wide surgical excision of a pathologically diagnosed basal cell carcinoma. The recurrence was diagnosed as infiltrative BCC and found metastasizing to skin, soft tissue and muscles, and pretracheal and axillary lymph nodes. Three cycles of chemotherapy comprising intravenous *cis*platin (50 mg) and 5-florouracil (5-FU, 750 mg) on 2 consecutive days and repeated at every 21 days were effective. As it remains unclear whether metastatic BCC is itself a separate subset of basal cell carcinoma, we feel that early BCC localized at any site perhaps constitutes a biological continuum that may ultimately manifest with metastasis in some individuals and should be evaluated as such. Long-standing BCC is itself potentially at risk of recurrence/dissemination; it is imperative to diagnose and appropriately treat all BCC lesions at the earliest.

## 1. Introduction

Basal cell carcinoma (BCC), a slowly progressive and poorly metastasizing skin cancer with propensity to be locally destructive, accounts for almost 80% of all nonmelanoma skin cancers worldwide [1]. Despite such a high prevalence its metastasis is extremely rare ranging between 0.0028 and 0.55 of all BCC cases [2]. Almost 85% of metastatic BCC arise from primary lesions in the head and neck region and is less frequent from BCC over back and extremities [3]. Metastatic BCC typically occurs in middle-aged men having BCC of long duration, and the spread in order of frequency is usually to lymph nodes, lungs, bones, skin, or to other sites. Giant BCC, a tumor of more than 5 cm at its largest diameter, is its rare and aggressive form and occurs commonly on trunk [4]. However, in a metastatic BCC the primary cutaneous tumor must have distant metastatic lesions with histopathologic features identical to the primary tumor [5]. The described case is of metastatic BCC over the back with recurrence 4 years after excision and metastasis to regional lymph nodes, skin, soft tissues and muscles.

## 2. Case Report

**Figure 1:**
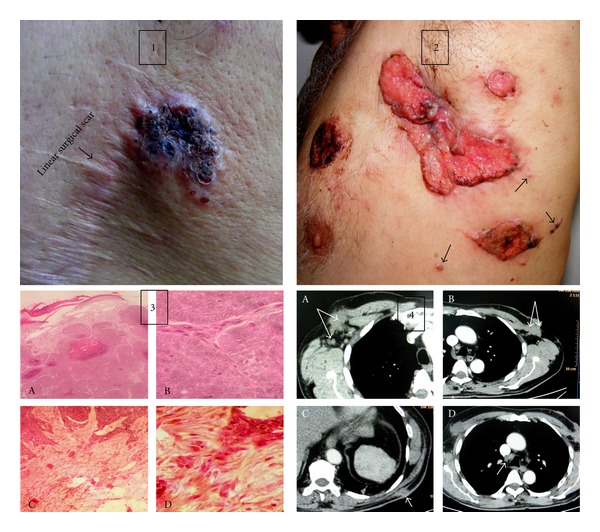
Panel 1: a nodulo-ulcerative basal cell carcinoma (BCC) plaque having characteristic indurated margins and pigmented crusts in the centre over back. Note the BCC lesion involving the margin of old linear scar. Panel 2: a large irregular nodulo-ulcerative fleshy plaque in left axilla. Puckering suggests adherence to other structures. Note typical beaded and pigmented borders and 3 morphologically similar lesions in its vicinity. Also note small satellite papulonodules indicative of local spread (arrow heads). Panel 3 (histology (A)): epidermis shows focal epidermal ulceration while dermis has numerous nests of basaloid cells (H and E, ×10). (B) The basaloid cells have increased mitotic activity and are arranged in peripheral palisading pattern with areas of central necrosis (H and E, ×40). (C and D) Tumor infiltrating the deeper layers is suggestive of infiltrative BCC (H and E, ×10 and ×40). Panel 4: CT scan (arrows) shows homogenously enhancing tumor infiltrating the underlying lymph nodes and muscles in right axilla (A) and left axilla (B). A subcutaneous tumor along the posterior axillary line (C). Enlarged lymph nodes in pretracheal region (D).

This 69-year-old male presented with multiple noduloulcerative lesions over upper trunk. History revealed that he had a nodule over his back that was diagnosed pathologically as basal cell carcinoma (BCC), and a wide excision was performed 6 years ago. During past 2 years the lesions had reappeared starting with the one over the old scar and were progressive in size and number. Cutaneous examination (Figure 1, Panels 1 and 2). showed a 3.0 × 2.5 cm noduloulcerative plaque with firm indurated base, elevated margins, and black crusts in the centre and involving the lateral part of a scar on his left midback. A large irregular shaped fleshy noduloulcerative plaque sized 10.0 × 3.0 cm having uneven surface, central puckering, and beaded and pigmented margins was present in the left axilla. Surrounding this plaque was other three noduloulcerative plaques of similar morphology which measured from 3 to 5 cms and few small papulonodular satellite lesions. Another large tumoral nodule sized about 3 cm on the left scapular area was firm in consistency and fixed to the underlying tissues. Axillary lymph nodes were enlarged bilaterally. They were firm in consistency and fixed to the overlying skin and deeper structures. The scalp, hair, nails, mucous membranes, and other systemic examination showed no abnormality. Complete blood counts, serum biochemistry, X-ray chest, abdomen ultrasonography, and urinalysis were essentially normal. Histologic examination of the nodule over the old scar revealed features of infiltrative BCC (Figure 1, Panel 3). Fine needle aspiration cytology from right axillary lymph node and a nodule over left scapular region was also suggestive of infiltrative BCC. Plain and contrast enhanced computed tomography (CT) (Figure 1, Panel 4) revealed homogenously enhancing masses, measuring 36 × 30 mm, and 41 × 33 mm and invading the underlying muscles both in right and left axilla, respectively. A soft-tissue mass of 18 × 17 mm was seen in the subcutaneous tissue along the left posterior axillary line, and enlarged lymph nodes were observed in pretracheal region and both axillae. There were no metastases noted in the pleurae, lung parenchyma, mediastinum, or underlying bones. He was put on chemotherapy comprising *cis*platin (50 mg) and 5-florouracil (5-FU, 750 mg) given intravenously for 2 consecutive days and repeated at every 21 days. Intravenous palonosetron (25 mg) and dexamethasone (8 mg) were used to prevent chemotherapy-induced nausea. All lesions progressively regressed in size after 3 cycles of chemotherapy which was well tolerated by him.

## 3. Discussion

Metastatic BCC remains a rare entity and affects males two times more often than females [6]. Age at presentation, the site and size of lesion, depth of invasion, duration and recurrence of disease, incomplete surgical resection, multiple lesions, and infiltrative histological pattern play some role in predicting metastasis [7]. The median age of onset for primary tumor is 45 years, and the median age at the time of metastasis is 59 years while the median interval between appearance of the primary tumor and metastasis is 9 years [2]. In 85% cases the metastasis occurs from the primary tumor situated in the head and neck (face) region and is infrequent from BCC over back and extremities [3]. Naso-cheek, paranasal and retroauricular folds, and inner canthus are considered to be critical sites, but there is no consensus as to whether any one histologic variety (basosquamous, micronodular, infiltrative, or morphea BCC) of the primary tumor has any predisposition for high rate of recurrence/metastasis [6, 8]. The incidence of metastasis is 2% for tumors larger than 3 cm in diameter; it increases to 25% for tumors larger than 5 cm in diameter and 50% for tumors larger than 10 cm in diameter [9]. Giant BCC, commonly occurs on trunk, is aggressively destructive and possesses high metastatic potential probably due to involvement of large blood vessels leading to seedling of tumor via hematological route and its spread [4, 10, 11]. Fair skin, trisomy of chromosome 6, immunosuppression in affected patients, invasion of perineural space and blood vessels, multiple recurrences, and prior radiation therapy are some of the other risk factors described for metastasis in BCC [12, 13]. Metastatic BCC most often manifests as dissemination to the regional lymph nodes (60%), lungs (42%), bones (20%), skin (10%), or to other organs in 2% cases via hematogenous spread or direct extension [14]. Metastasis to liver, other viscera, or subcutaneous tissues may occur following involvement of lymph nodes, lungs, or bones. Primary tumors may also invade deep into the extradermal structures such as cartilage, skeletal muscles, or bones. However, according to Lattes and Kessler [5] to label a metastatic BCC the primary lesion must occur in the skin and not in a mucous membrane, metastasis must be at a site distant from the primary tumor and not merely a simple extension, and both primary and metastatic tumors must have similar histopathology. Although we have no recorded information about the exact size of the primary tumor, it must have been >5 cm as is evident from the size of postexcision scar; it had left. All other features in our patient who had developed BCC at the age of 59 years, its localization over skin, recurrence of multiple lesions over trunk 4 years after its excision, metastasis to soft tissue and regional lymph nodes away from the primary site, and infiltrative histological pattern of both primary and metastatic lesions conforms to the diagnostic criteria proposed by Lattes and Kessler [5]. However, it seems that it is not necessarily the BCC over head and neck region that gets metastasized as has been suggested by Malone et al. [3], BCC over other sites such as back as in our patient appears equally prone to metastasis. Invasion of the underlying muscles in him too is a rare occurrence. 

 Therapy of metastatic BCC depends upon the location and extent of the tumor and generally consists of wide surgical excision alone for local metastasis or its combination with chemotherapy and radiation therapy for distant metastasis. The main aim of the surgery is complete excision of the tumor with clear margins. The recurrence rate following surgical excision varies between 5% for complete excision and 30% for excisions with positive margins [14]. Mohs' micrographic surgery is the treatment of choice for histopathologically aggressive BCC subtypes owing to low recurrence rate, it has shown [8]. Several chemotherapeutic agents including 5-FU, *cis*platin, vincristine, etoposide, bleomycin, cyclophosphamide, methotrexate, and doxorubicin have been used alone or in combination [15–17]. Although Pfeiffer et al. [15]  found *cis*platin to be the most effective chemotherapeutic agent, a combination regimen of vincristine, bleomycin, and prednisolone has been found more useful by others [16]. Our patient showed regression of tumor after 3 cycles of chemotherapy with *cis*platin (80–100 mg/M^2^) and 5-FU (600 mg/M^2^) combination.

The prognosis for metastatic BCC generally remains poor, and average survival time is variable, 8 months in the presence of distant metastasis, 3.5 years for patients with disease confined to lymph nodes, and several years as an exception [9, 18]. While overall metastatic risks of BCC perhaps remain underestimated, metastatic BCC for its rarity remains a difficult entity to characterize in terms of etiology, risk factors for metastasis, and therapeutic options. As most cases have been diagnosed retrospectively, it remains unclear whether metastatic BCC constitutes a separate subset of basal cell carcinoma or not. It is even considered a complication of BCC with high morbidity and mortality by some workers [14]. We feel that early localized BCC perhaps constitutes a biological continuum that may ultimately lead to metastatic BCC in some individuals, and it must be evaluated as such. Nevertheless, it is imperative to diagnose and treat all basal cell carcinomas at the earliest in view of paucity of knowledge on patient specific risk factors for metastasis, and the fact that a BCC neglected for longtime is potentially at risk of metastases. Vismodegib (GDC-0449), a hedgehog signaling pathway inhibitor, has been approved recently by USFDA for treating patients with BCC which can not be treated with surgery or radiation and has recurred after surgery or metastasized. Although in a recent multicenter nonrandomized study of vismodegib [19], the response rate was 30% in 33 patients with metastatic BCC and 43% in 63 patients with locally advanced BCC in a median duration of 7.6 months; serious adverse events including deaths were noted in 25% cases implying that one needs to be prudent pending more evaluation studies. For lack of consensus on chemotherapy protocol in widespread disease, inconsistent therapeutic outcome, and inadequately excised BCC being a possible cause of recurrence itself, the most expedient preventive measures for recurrence/metastasis include a thorough and complete surgical excision of the primary tumor itself.
